# Eye-Tracking in Interpreting Studies: A Review of Four Decades of Empirical Studies

**DOI:** 10.3389/fpsyg.2022.872247

**Published:** 2022-06-27

**Authors:** Ting Hu, Xinyu Wang, Haiming Xu

**Affiliations:** ^1^School of English Studies, Shanghai International Studies University, Shanghai, China; ^2^Zhejiang Conservatory of Music, Hangzhou, China

**Keywords:** eye-tracking, interpreting studies, empirical, review, eye movements

## Abstract

It has been four decades since eye-tracking was first used in interpreting studies, and recent years has witnessed a growing interest in the application of this method, which holds great potential for offering a look into the “black box” of interpreting processing. However, little attention has been paid to comprehensively illustrating what has been done, what can be done, and what needs to be done with this method in this discipline. With this in view, this paper sets out to understand contributions of previous studies—key themes discussed, eye-tracking measures used, their limitations and implications, and future directions. To this end, we conduct a review of a total of 26 empirical papers from peer-reviewed journals within a time span of 4 decades ranging from 1981 to 2021. This study, as the first attempt of its kind at a comprehensive review on using eye-tracking in interpreting studies, should have implications for researchers, educators, and practitioners.

## Introduction

Interpreting is a form of translation where an immediate and singular production in a target language is produced based on the one-time presentation of an utterance in a source language (Kade, 1968; Pöchhacker, 2016). Since Interpreting Studies (IS) take interpreting as its object of investigation, it is seen as a subdiscipline of the larger domain of Translation Studies (Pöchhacker, 2010). Salevsky (1993) classifies IS into the theoretical, descriptive, and applied domains, which covers topics including process, product and performance, practice and profession, and pedagogy (Pöchhacker, 2016). Among them, process research, that is, studies on the identification, decoding, transferring, and producing of linguistic units from one language to another (Nida, 1964), is a dominant theme in empirical IS (Pöchhacker, 2016). According to Gile (2009a) effort model, changes in the processing load of interpreters have an impact on the product; therefore, it is of high relevance to investigate and understand the process.

As interpreting is a complex linguistic activity (Hale, 2010; Stachowiak-Szymczak, 2020) and researchers do not have immediate access to interpreters’ brain, they resort to various methods to look into the “black box” of interpreting processing. These methods can be classified as offline or online ones. Offline methods refer to methods that are temporally separate from the main task, usually after it (Godfroid, 2019). Such an offline method is retrospective protocol, popular in interpreting studies (Dimitrova and Tiselius, 2014), boasts operational convenience, and yields qualitative data on the cognitive mechanisms behind certain phenomena, thereby providing answers to “why” they take place (Dimitrova and Tiselius, 2009). However, retrospective protocols are prone to subjectivity and lower accuracy, as there may be a gap between what is perceived or reported and what really happened (Ericsson and Simon, 1984; Ivanova, 2000). Moreover, offline methods fail to capture the moment-to-moment interpreting process and may miss crucial details of it.

Online methods, in contrast, enable the concurrent, real-time observation and collection of data during the task under study (Godfroid, 2019). They primarily include two subcategories: performance methods, such as analysis of ear-voice span (EVS), disfluencies, and omissions, and psychophysiological methods, such as eye-tracking, event-relatesd potential (ERP), and positron emission tomography (PET) (Tirkkonen-Condit and Jääskeläinen, 2000; Timarová, 2010; Seeber, 2013; Godfroid, 2019). Between these two subcategories, performance data are more readily available, as collection and analysis of them usually do not require additional and complicated apparatuses. For instance, researchers have easy access to performance data with recordings of the interpreting tasks. But compared with psychophysiological methods, such as functional magnetic resonance imaging (fMRI) that provides information on the process- brain activities, performance methods are more subjective, as researchers need to infer the interpreting process from the product. According to Seeber (2013), psychophysiological methods are more objective, since they record ocular, cerebral, and other physiological responses, which cannot be consciously controlled. Furthermore, they generate concurrent data that allow for moment-to-moment investigation of interpreting processes. With some psychophysiological methods, however, the trade-off for these merits is low ecological validity, that is, the representativeness or naturalness of experimental situations, materials or task, or how much they resemble the actual, real-life conditions (Kvavilashvili and Ellis, 2004; Spinner et al., 2013). Since the closer the resemblance, the more generalizable findings from the interpreting study can be, high ecological validity is usually a desirable feature (Kvavilashvili and Ellis, 2004). But such a virtue is hard to come by for some psychophysiological methods. For example, in studies with ERP, participants need to stay stationary, but real-life interpreting often involves head movements, hand gestures, or even body movements of interpreters. Therefore, the constraints posed on interpreters by ERP apparatus would influence the interpreting process and make it unnatural, or less authentic.

Among popular psychophysiological methods in IS, such as eye-tracking, PET (Rinne et al., 2000; Tommola et al., 2000), ERP (Proverbio et al., 2004; Koshkin et al., 2018), and fMRI (Hervais-Adelman et al., 2011; Becker et al., 2016; Van De Putte et al., 2018), eye-tracking has the advantage of higher ecological validity, high temporal and spatial accuracy and easier operation (Godfroid, 2019; Tiselius, 2020). To start with, the emergence of more flexible and less intrusive eye trackers, such as eye-tracking glasses, allows for movements of the interpreters (Mele and Federici, 2012). Therefore, it is possible to document eye movements in a more normal or actual state of interpreting. Secondly, eye trackers offer high temporal resolution of milliseconds and high spatial resolution at the sub-lexical level (Godfroid, 2019). Another advantage of eye-tracking over other psychophysiological methods is its operational convenience, such as instant configuration, execution, and calibration (Conklin and Pellicer-Sánchez, 2016). Taken together, it is a non-intrusive, relatively easy-to-operate method that allows for real-time, fine-grained investigation into the dynamics of interpreting processing (Hvelplund, 2014; König et al., 2016; Tiselius, 2020).

By definition, eye tracking refers to “an experimental method of recording eye motion and gaze location across time and task” (Carter and Luke, 2020, p. 50) that provides significant information about the operation of the human brain (Rayner, 1978; Leigh and Zee, 2015). Eye movements generally include fixations, saccades, and other types of ocular motion such as vergence and pupil dilation or constriction (Carter and Luke, 2020). The hypotheses that link eye movements to cognitive activities are Just and Carpenter’s (1980) eye-mind and immediacy assumptions, which postulate that what is being fixated is what is being processed, and there is no appreciable lag between the two. Despite limitations of these assumptions, for example, they cannot explain the phenomenon of mind wondering independent of eye movements (Shepherd et al., 1986; Smallwood and Schooler, 2006), they are regarded as the theoretical underpinnings of eye-tracking (Seeber, 2015). The history of eye-tracking studies dates back to the 1870s, when Javal first identified two major types of eye movements during reading: fixations and saccades (Yan et al., 2013; Płużyczka, 2018). The method first became popular in psychology and psycholinguistics, data from which were seen as “the gold standard for experiments” (Rayner, 2009, p. 1474). It was then introduced in IS by McDonald and Carpenter (1981), who investigated the interpretation and parsing of ambiguous idiomatic phrases, as well as error detection and correction in sight translation, a variant of interpreting (Herbert, 1952; Agrifoglio, 2004; Chen, 2015; Pöchhacker, 2016). Later, it was increasingly adopted in linguistics, as evidenced by a growing number of studies in this field using eye-tracking in the 21st century (Conklin and Pellicer-Sánchez, 2016), and is now gaining ground in IS (Korpal, 2015; Ma, 2017; Tiselius, 2020).

However, despite the increasing popularity of this method in IS, research that delineates a comprehensive and systematic picture of eye-tracking in IS seems to be lacking. We argue that it is necessary to have a clear and comprehensive overview of what can be done with this method, what has been done, and what still needs to be done. With this landscape of eye-tracking interpreting research, researchers can better understand how their research fits into it. This is especially valuable for researchers unfamiliar with this method, and aiming to explore new territories with eye-tracking in IS. Moreover, it is of theoretical relevance to conduct a synthesis on current interpreting studies with eye-tracking and on findings from them. For instance, these findings can offer valuable information on the underlying interpreting mechanisms, and can be applied in building new interpreting models or validating existing ones, such as Gile’s (2009a) Effort Model. In addition, these findings, for example, on eye movement patterns in case of difficulties, in the employment of specific strategies, and their differences between trainees and professionals also contribute to interpreting training. Despite the insights afforded by previous reviews of eye-tracking IS, they are somewhat limited in breadth and scope, as the focus is either several selected studies (Moratto, 2020), or a specific research dimension—methodological issues (Korpal, 2015), or a specific theme—cognitive load (Su et al., 2021), or a specific interpreting mode—sight interpreting (Ma, 2017). Thus, this is the first attempt at a comprehensive review of eye-tracking in IS in its entirety. With this review, we aim to identify common practices and problems, as well as explore the potential of eye-tracking in IS. Specifically, we (1) review publication trend of previous studies; (2) analyze the design features of these empirical papers, including participants, interpreting modes, and eye trackers used; (3) summarize their contributions—key themes discussed and eye-tracking measures used; (4) discuss the limitations and their implications, and (5) propose potential avenues for future research. This is of academic, theoretical, and practical relevance, as it can help researchers get an overview of extant literature, design future studies, and gain insight on what eye-tracking stands to offer, deepen our understanding of interpreting mechanisms, and inform interpreting training, for example, through identification of interpreting difficulties and differences of eye movement patterns between professionals and trainees.

## Materials and Methods

This study follows Cooper’s (2016) comprehensive and widely adopted framework of research synthesis with the following stages: formulating the problem, searching the literature, gathering information form studies, evaluating the quality of studies, analyzing and integrating the outcomes of studies, interpreting the evidence, and presenting the results. Since the aim of this study is to identify how eye-tracking is employed in IS, empirical articles are selected, and to ensure the representativeness of data, we choose peer-reviewed journals as the data source.

### Paper Selection

**Figure 1:**
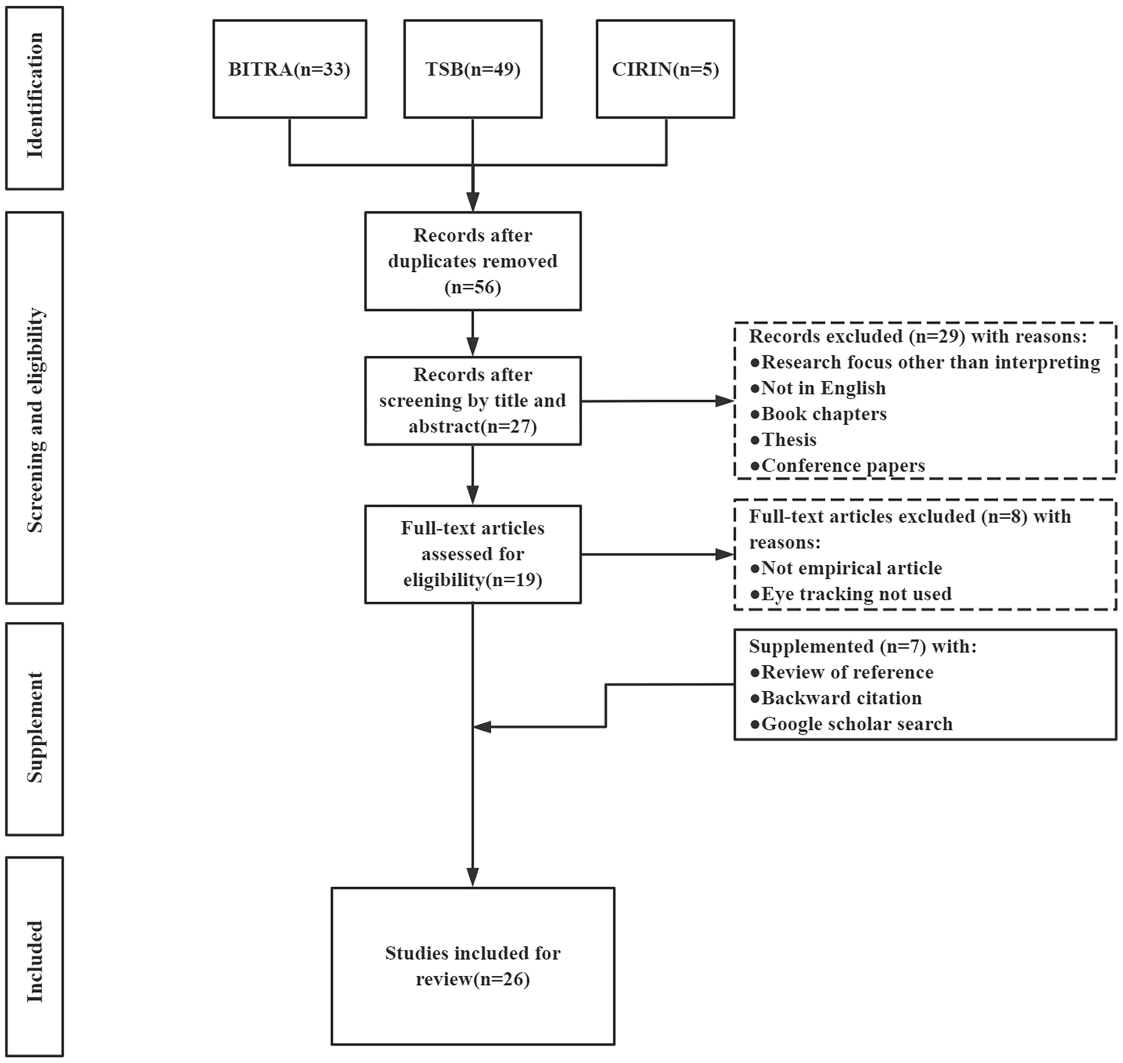
Flow diagram depicting paper selection procedure.

Figure 1 illustrates the paper selection procedure. Three key databases for interpreting and translation studies were searched as the first step: Bibliography of Interpreting and Translation (BITRA), Translation Studies Bibliography (TSB), and Conference Interpreting Research Information Network (CIRIN). A combination of the following keywords was used: “interpreting,” “sight translation,” “eye movement,” “eye-tracking,” “gaze movement,” and “gaze tracking” (paper selection was conducted in July 2021). The data pool was then screened manually with five inclusion criteria: (1) Article was published in peer-reviewed journals, with the exclusion of book chapter and conference paper; (2) Eye-tracking was used in the study; (3) Article was on interpreting research; (4) Article was an empirical research paper, with the exclusion of book review, commentary; and (5) Article was published in English. To make the review more inclusive, we did not restrict language pair or directionality, thus interpreting studies on a variety of language pair and directions were included. In the third step, to ensure exhaustiveness of results, results from the previous step were supplemented by another round of searches through review of reference and backward citation of eligible articles, as well as Google Scholar search with the same keywords combination of step one. Finally, 26 papers were identified as data pool for this review.

### Coding Scheme

A three-stage thematic coding was done for analysis by the first author and another researcher with rich experience in eye-tracking studies. In the initial stage, we scan through the abstract, introduction, discussions, and conclusions of retrieved papers together for the research foci, and decided five thematic dimensions: cognitive load, cognitive processing, utilization of visual input, viewing patterns, and the role of gaze. In the second stage, the first author explained guidelines for coding and the two coders were then allowed 1 week to read them in-depth and code 26 articles separately. An inter-coder reliability analysis using the Cohen’s kappa was performed to determine consistency among coders, and a high agreement was found: kappa = 0.869 (*p* < 0.001), 95% CI (0.732, 1.000). Finally, coders tried to solve the three disagreements through in-depth discussion. Together, the two coders reread the papers where disagreements emerged, and the second coder was persuaded by the first on two incongruent codes, and for the one remaining incongruency, the first coder took into account the second’s suggestions and revised the code. For example, the second coder took utilization of visual input as the research focus of Bosch-Baliarda et al.’s (2020) paper; however, after discussion, its themes were coded as both utilization of visual input and viewing patterns, as number and duration of fixations, visits, and heatmaps were examined also for behavioral patterns (see Appendix for detailed information on coding results).

## Results

### Publications

**Figure 2:**
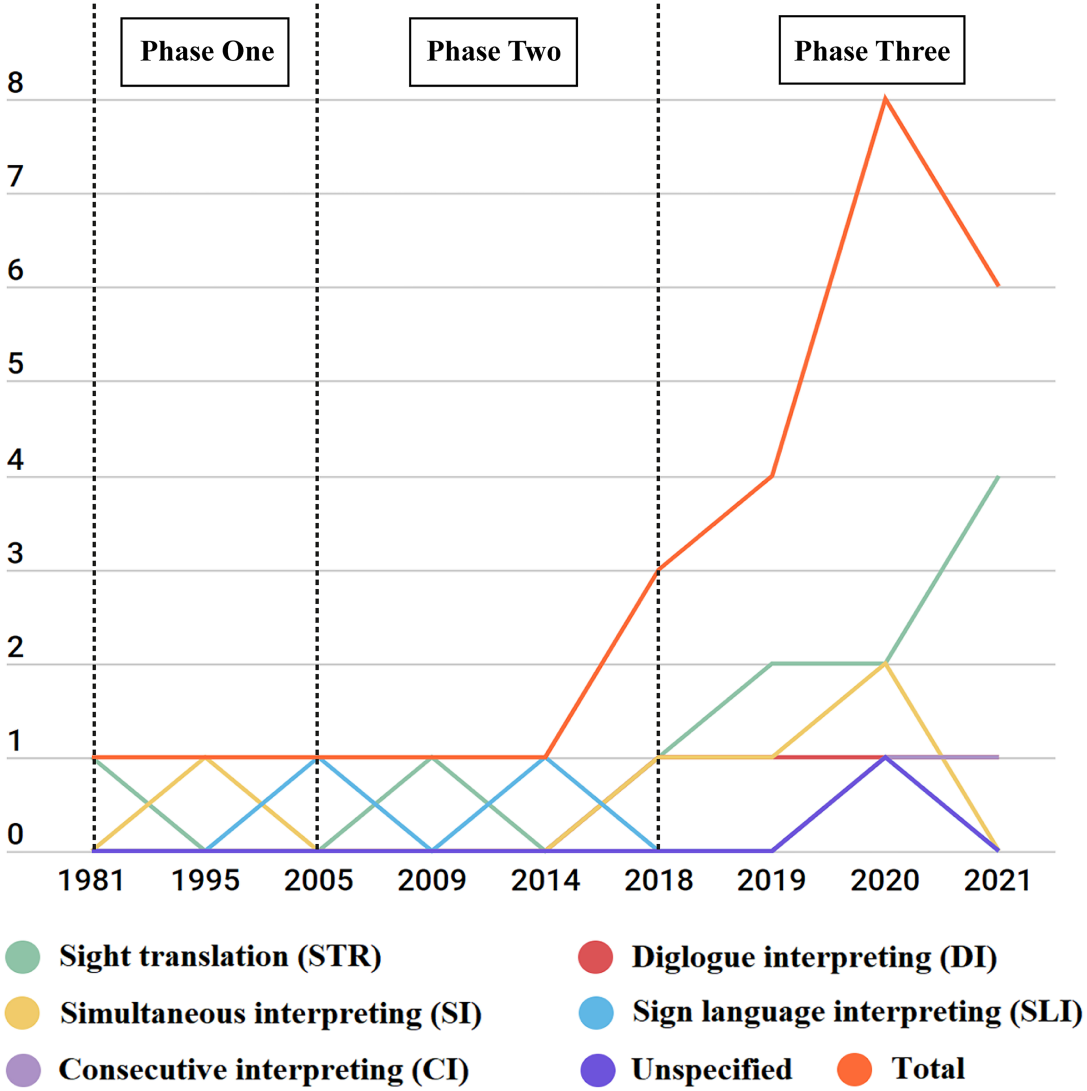
Phases of publication.

As can be seen in Figure 2, the orange line depicts the growth trajectory of the number of published peer-reviewed empirical articles of interpreting research with eye-tracking. Based on the number of publications and authors’ scholarly background, we divide publication of IS with eye-tracking into three phases. The first phase (1981–2004) was characterized by a small number of publications (two papers in total), mostly studies initiated by psychologists (e.g., Jukka Hyönä, Patricia A. Carpenter). This period came in the wake of the establishment of a theoretical and methodological basis of cognitive psychology in the 1970s and coincided with a strong influence on interpreting studies from cognitive science (Gile, 2009b; Płużyczka, 2018). Therefore, scholars such as psychologists took an interest in interpreting, and attempted the application of eye-tracking to IS (McDonald and Carpenter, 1981). SI was also used as a highly complex task to test the reliability of pupillometry as an indicator of cognitive load (Tommola and Hyönä, 1990; Hyönä et al., 1995). In the second phase (2005–2017), publications were still few and far between (three papers in total), but it was during this period that translation and interpreting scholars began to employ eye-tracking in IS (e.g., Barbara Dragsted, Jennifer Wehrmeyer), and publications mostly came from these researchers. This phase witnessed the increasing availability of wearable, portable, wireless, and real-time streaming eye gaze trackers on the market (Cognolato et al., 2018), and for this reason, eye trackers began to attract the attention of translation and interpreting researchers to study interpreting activities. However, eye-tracking systems during this phase were still not sufficiently developed to meet both needs of tolerating participants’ head movements and a high sampling rate, an indicator of data quality (Conklin et al., 2018). This can be seen from the mobile eye trackers with low sampling rate (60HZ) used in the studies from this period (Marschark et al., 2005; Wehrmeyer, 2014). As a result, such eye trackers would be unreliable for research with small interest areas, such as on lexical processing during interpreting. Then eye-tracking in IS entered the third phase (starting from 2018), which was marked by more publications from 2018 onwards, thanks to continued technological developments, especially advances in eye tracking technologies (Cognolato et al., 2018). For instance, Tobii Pro Spectrum (at 1200 Hz), a type of remote, head-free-to-move eye tracker with a sampling rate of up to 1200 Hz, was launched at the end of 2017 (Tobii, 2017). Such an apparatus not only yields high quality data, but also makes the interpreting experiments more resemblant to the actual interpreting, as there are few constraints on the participants, for example, from putting their heads on the head-rest, as seen in head-stabilized eye trackers. In other words, the more advanced models make it possible for researchers to conduct research with high ecological validity and high data quality. Apart from the constraints on interpreting participants’ movements and the resulting lower ecological validity coming from using older models of eye trackers, another reason that interpreting researchers refrain from applying eye trackers is their high prices (Keating, 2014). But, the future will see wider use of eye-tracking in IS, since with sustained technological advancement, better and more affordable eye trackers will hit the shelves.

### Design Features

#### Participants

On average, 24.88 participants were recruited in the studies (SD = 14.18, Min = 3, Max = 55). However, data from some participants had to be excluded for analysis, due to reasons varying from their body movements, to failures in calibration, and to data failing to meet quality criteria (Wehrmeyer, 2014; Ma and Li, 2021). The data attrition rate in the studies ranged from 2.78 to 32%, which is to be expected, as the data loss rate reported by studies can lie between 2 to 60% (Holmqvist et al., 2012). Therefore, it is advisable for researchers to enlist more participants than is necessary, as data loss is common in eye-tracking studies.

**Figure 3:**
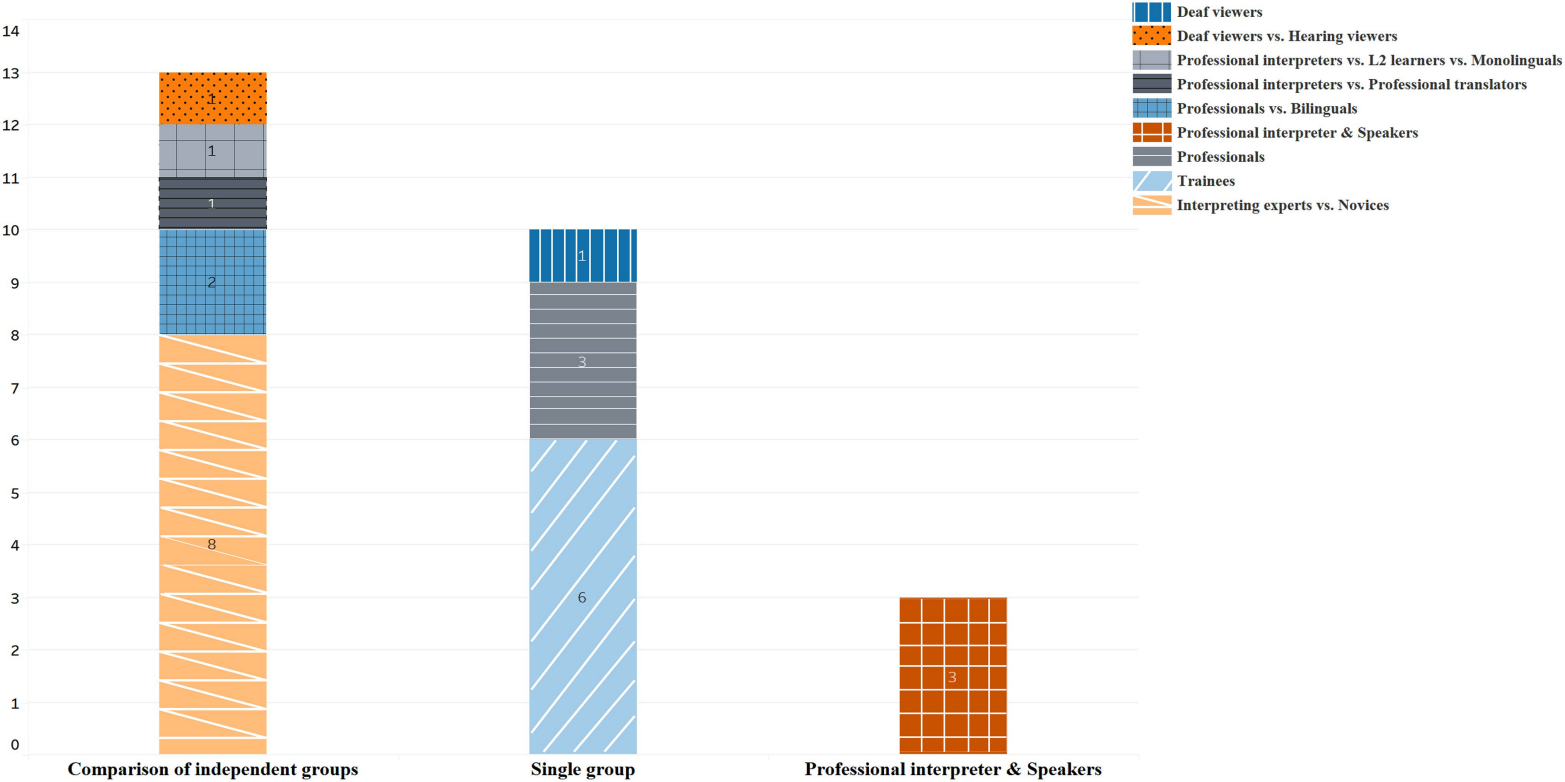
Number of papers for each type of participants paradigm.

As is shown in Figure 3, the most common design (found in 13 studies; 50%) in the reviewed studies is the comparison of independent groups. Among them, the most popular comparison is the expert-novice paradigm (8;31%), in accordance with findings from previous research (Timarová, 2010). Since professional and trainees were found to differ in both declarative knowledge and procedural knowledge, in the form of factual, semantic, and schematic knowledge, as well as strategic knowledge (Moser-Mercer, 1997), such comparison allows researchers to pin down what and how they are different from a cognitive and behavioral perspective. In so doing, pedagogical information can be gleaned. Three studies simulated real-life interpreting and recruited both speakers and professional interpreters. For the rest 10 studies, one group of participants were recruited, six of them enrolling trainees only, three of them professionals only, and one deaf viewer only. In the six studies with trainees as participants, some endeavored to follow the longitudinal development of trainees’ interpreting abilities, and some enrolled trainees as a substitution for professionals. The latter situation is understandable and is often the case with IS, as it is difficult to recruit professionals. However, it should be noted that findings from trainees do not always apply to the larger community of interpreters. This is because interpreting trainees are hardly representative of interpreters, as their interpreting skills, for instance, fall short of the standards of professionals (Gile, 2015), and also as explained earlier, differences exist between the two groups in declarative and procedural knowledge (Moser-Mercer, 1997). It is also worth noting that researchers can design a valid and sound empirical study with a limited number of participants. For example, Vranjes et al. (2018) studied dialog interpreting with only three participants: two speakers and one professional interpreter. However, this qualitative case study managed to offer in-depth knowledge about the coordination of listener responses and gaze in this interpreting activity.

#### Interpreting Modes

**Figure 4:**
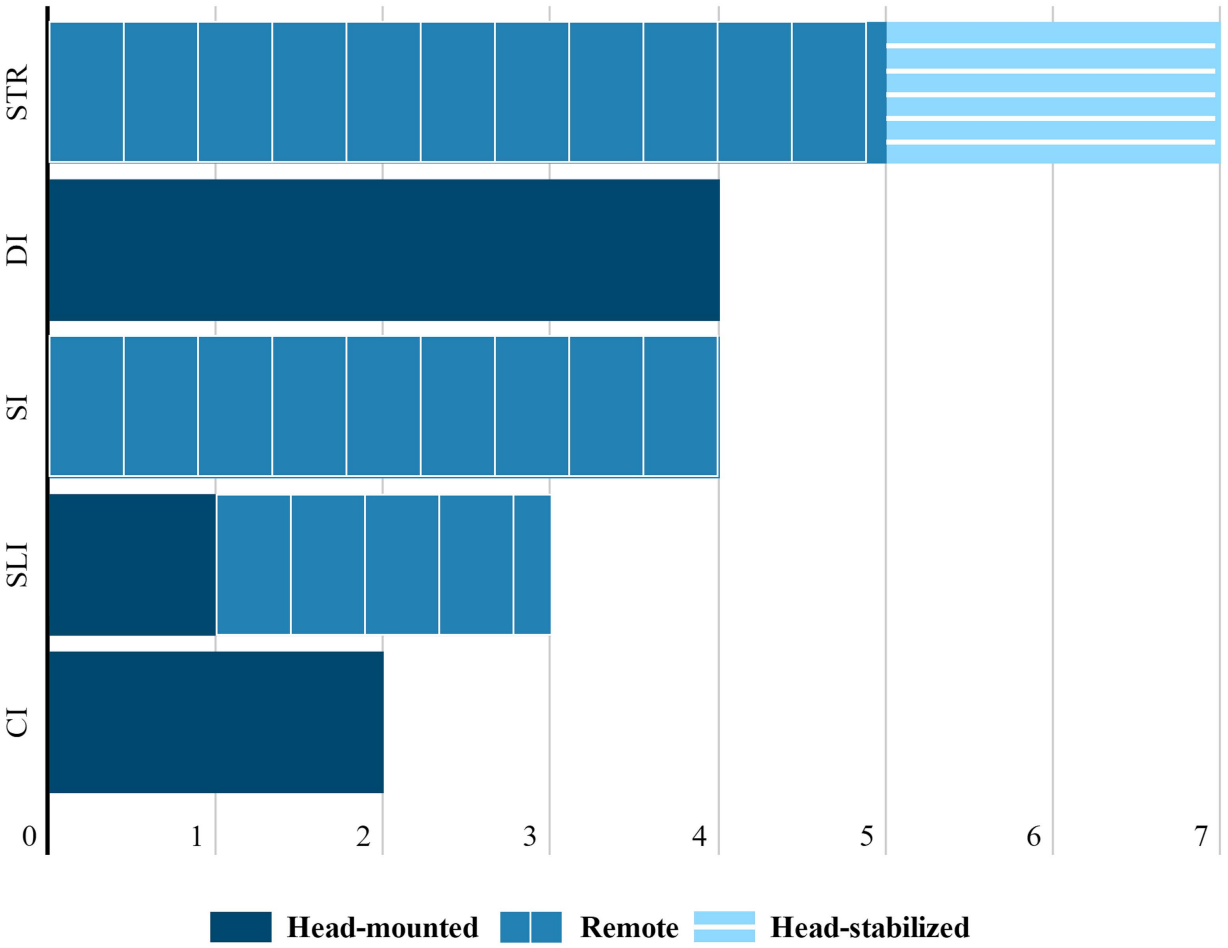
Choice of eye trackers for investigation of different interpreting modes.

The use of eye-tracking in interpreting studies has been on the rise in recent years, as is shown in Figure 4. Among different interpreting modes, sight translation (STR) was the most investigated one with eye-tracking (11;42.31%). STR is the oral rendition of a written text (Herbert, 1952; Agrifoglio, 2004; Pöchhacker, 2016). The reason that STR is studied with eye-tracking more than other modes of interpreting is probably that most of, if not all of, the source information comes from the visual input. But, recent years has also witnessed the application of eye-tracking in other modes: consecutive interpreting (CI), a mode where interpretation comes after the source-language utterance; simultaneous interpreting (SI), a type practiced as the source text is uttered; dialog interpreting (DI), where interpreters work back and forth between source and target languages; and sign-language interpreting (SLI), which involves the rendition between the two language modalities of spoken language and sign language (Pöchhacker, 2016). For example, Chen et al. (2021) brought a new perspective to note-reading of CI by visualizing it through semantic gaze mapping. Noticeably, there seems to be renewed interest in using eye-tracking in SI studies, more than two decades after eye-tracking was first used in the seminal work by Hyönä et al. (1995) to verify the reliability of pupil dilation as an indicator for processing load in SI and other language tasks. In the SI studies reviewed, eye-tracking was mainly applied to explore cognitive processing with the presence of both visual and aural information input. An example of this is the study carried out by Korpal and Stachowiak-Szymczak (2020) in which they examined oculomotor behaviors, such as scanning and fixations, to PowerPoint presentation slides and numbers on them during SI, of both professional interpreters and interpreting trainees, and at both fast and slow delivery rate.

#### Eye Trackers

Adopting an appropriate apparatus is vital to quality eye-tracking research, as it decides the feasibility of conducting a study in the first place (Holmqvist et al., 2011). In line with Hvelplund’s (2014) and Conklin et al.’s (2018) taxonomy, there are mainly 3 types of eye trackers: a) high precision, head-stabilized eye trackers; b) remote, head-free-to-move eye trackers; and c) head-mounted eye trackers. High precision, head-stabilized eye trackers (e.g., Eyelink 1,000 Plus) are the ones that come with a head support, for instance, a chin rest, to keep the head stable (SR-Research, 2022). By contrast, remote and head-free-to-move eye trackers (e.g., Tobii Pro Spectrum at 600HZ) allow for head movement to a larger extent (Tobii Pro, 2022). Head-mounted eye trackers (e.g., SMI Eye Tracking Glasses, or SMI ETG), devices worn on the head, are more tolerant to head movements than foregoing types but are more invasive, as participants have to wear them on the head (Hvelplund, 2014; Conklin et al., 2018; Godfroid, 2019; Imotions A/S, 2022).

As Figure 4 illustrates, among the reviewed studies that reported specific types of eye trackers, remote eye trackers were generally found to be the most popular (11;55%), consistent with Hvelplund (2014) proposal for choosing this type in translation studies. Remote eye trackers were also the likeliest choice for STR, SI, and SLI studies. A major advantage of them is that they allow for a certain degree of head movement while featuring a relatively high sampling rate, which offers more fine-grained information with data of higher quality. This dovetails with the need for occasional head movements of simultaneous interpreters, sight translators, and deaf viewers of SLI. Head-stabilized eye trackers were the least chosen type (3;15%). Their high precision notwithstanding, they were not found to be used in any mode other than STR. This is understandable, because for other types of interpreting, including CI, SI, DI, or SLI, head or body movements are more or less involved. Therefore, it is quite difficult to study these interpreting types with head-stabilized eye trackers, which confine the movements of the participants’ heads by keep them steady on a chin-rest or head-rest. But they are most advantageous when a high sampling rate is required and little head movement is involved, such as in STR studies with higher requirements for data quality. We also found that head-mounted eye trackers, mainly eye-tracking glasses in the reviewed studies, were preferred to investigate CI and DI. One prominent advantage of head-mounted eye trackers over other types is that the recording area of the device is not restricted to the computer monitor (Hvelplund, 2014). In CI or DI, Interpreters’ heads move more drastically, constantly shifting from the speaker to notes in CI, or from one speaker to another during DI. From this perspective, eye-tracking glasses afford a more natural research process of CI and DI, as they allow for interpreters’ free interaction with the environment. But the trade-off for their flexibility is a lower sampling rate and less accurate data than the above-mentioned two types, which means reliability would suffer (Kredel et al., 2017). In other words, researchers need to seek a balance between ecological and external validity of experimental conditions and methodological reliability. In general, the studies reviewed chose eye trackers in accordance with their research questions, sizes of areas of interest, and properties of interpreting tasks. However, one limitation emerging from the studies reviewed is that while the papers report eye trackers’ types and model, not all of these studies described the important information of eye trackers’ specifications. For example, 11 out of the papers reviewed (42%) neglected to specify eye trackers’ sampling rate.

### Themes

**Figure 5:**
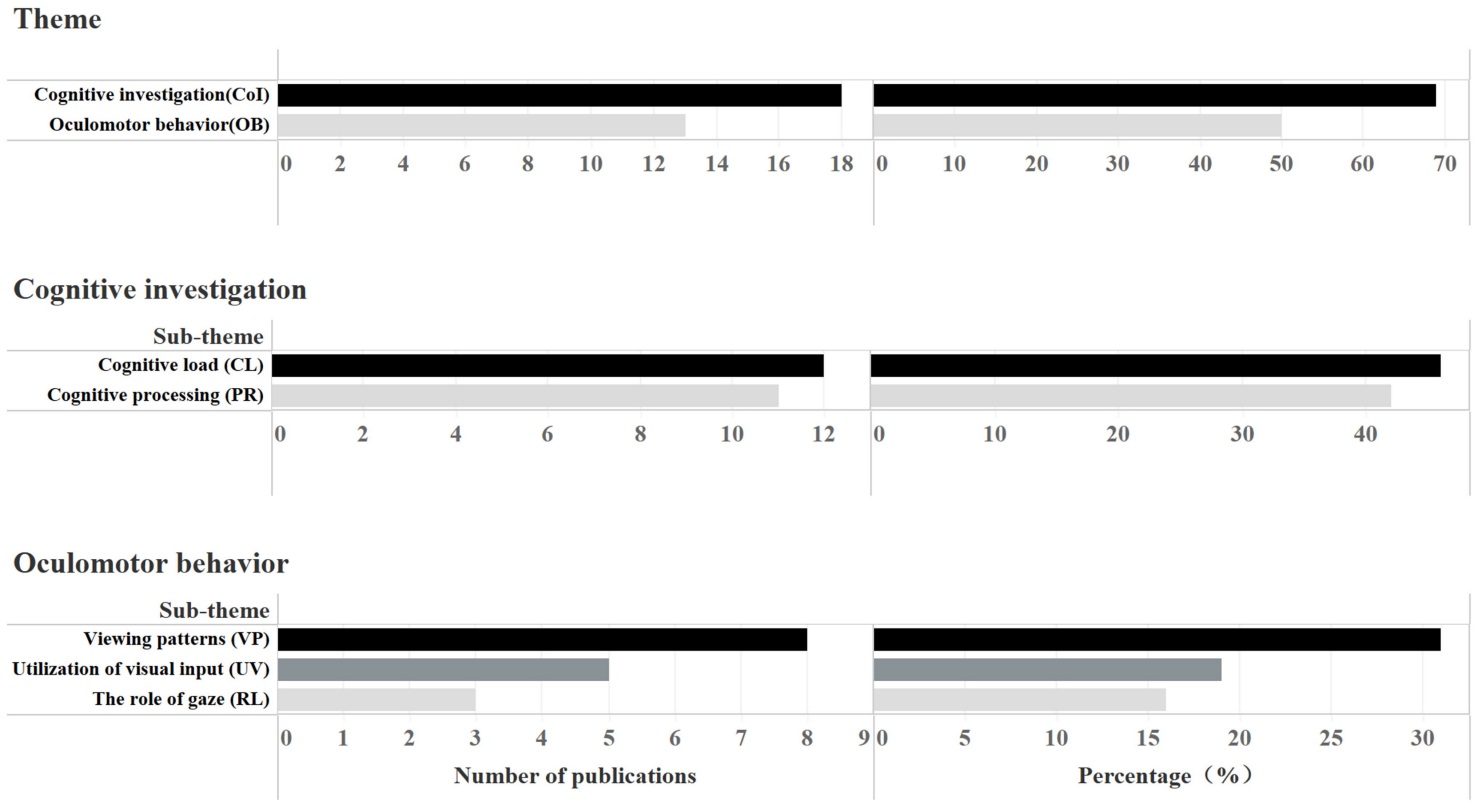
Number and proportion of papers investigating each theme and their sub-themes.

The questions that reviewed papers tried to answer with eye-tracking can be categorized into two overriding themes: higher-level cognitive and linguistic factors, and lower-level visual and oculomotor factors. Cognitive investigation (CoI) mainly deals with what is going on inside interpreters’ brains, the internal and hidden side of interpreting. By contrast, oculomotor behavior (OB) is concerned with the physical, external and observable side of it (Godfroid, 2019). These two themes are interconnected, as oculomotor behaviors can be the manifestation of cognitive activities, and cognitive activities can be inferred from oculomotor behaviors (Rayner, 2009). We made a distinction between them in this review as we found that for some papers, eye-tracking is used ultimately to examine the cognitive aspects of interpreting, while for others, it is used to explore eye movements *per se,* such as interpreters’ allocation of visual attention on different visual input. Therefore, the reviewed papers’ principal research purpose was adopted as our standard for labeling the two themes in the coding process. In light of this, studies with a predominant focus on the cognitive investigation were labeled as CoI, on oculomotor behavior, as OB, and studies that addressed both themes were labeled as CoI/OB. Figure 5 presents the number and proportion of these two themes, as well as sub-themes under them (since some papers simultaneously investigate several themes, their aggregated percentage exceeds 100%, see detailed coding of themes in Appendix).

#### Cognitive Investigation

One noteworthy contribution of eye-tracking is that it sheds light on the “black box” of interpreting: cognitive mechanisms. A total of 18 studies (69%) in the papers reviewed were found to conduct cognitive investigation with eye-tracking, and among them, two sub-themes can be identified: (1) cognitive load (CoI-CL); and (2) cognitive processing (CoI-PR; Figure 5).

##### Cognitive Load

Eye-tracking has proved to be instrumental for indexing cognitive load in interpreting studies, and a large proportion of studies reviewed (12;46%) have explored this theme. This method yields pupillometric and fixation data, commonly used indicators of cognitive load in interpreting studies (Hyönä et al., 1995; Hvelplund, 2014), thus offering insight into understanding the nature of interpreting. As a kind of psycho-physiological method, eye-tracking gains an advantage over other major methods for cognitive load assessment, such as analytical methods, subjective methods, and performance methods, because it is more objective, and enables moment-to-moment analysis of the process (Paas et al., 2003; Schultheis and Jameson, 2004; Seeber, 2013). Moreover, compared with other psycho-physiological methods, such as electroencephalography (EEG) or positron emission tomography (PET), it is more affordable and less intrusive (Seeber, 2013).

Among the papers reviewed, Hyönä et al.’s (1995) landmark study demonstrated the effectiveness of pupillary responses as indicators of cognitive effort. In this methodological paper, they conducted three experiments where participants were asked to finish different types of language tasks with varying degrees of difficulty, for instance, listening, shadowing, and simultaneous interpreting, and the same task with different difficulty levels. Results showed increased pupil dilation in more difficult tasks across the three experiments.

Since then, researchers have adopted eye-tracking to evaluate cognitive load in different interpreting directions, in the presence of different problem triggers, and with different interpreting strategies. Directionality is one of the oldest and most debated issues in interpreting studies (Déjean le Féal, 1998; Gile, 2005), and discussions on whether to interpret from L1-to-L2 or L2-to-L1 have yielded inconclusive results. Eye-tracking lends researchers a new perspective to investigate this issue and allows them to compare the cognitive load of opposite directions in a more objective and natural manner, and ultimately offer insight on interpreter training, for example, on syllabus design. Chen’s (2020) study compared the cognitive load of the two opposite directions of the comprehension phase and the ensuing speech production phase in CI. With fixation data, ear-pen-span data, and analysis of interpreters’ notes, she found that L1-to-L2 direction induced lower cognitive load in the first phase and conversely, more cognitive effort in the second phase. In Su and Li’s (2019) study, the overall cognitive load was found to be higher in L1-to-L2 STR than the other way round. Tiselius and Sneed (2020) found interpreters gazed at no face more often, in other words, averted gaze more, during L1-to-L2 DI, and postulated higher cognitive load in this direction. In SI of single words, Hyönä et al. (1995) revealed increased pupil dilation in L1-to-L2 direction, implying a higher cognitive load. However, up till now, there has been no bidirectional comparison of cognitive load with eye-tracking in SI of larger linguistic units, such as at syntactic or discoursal levels. It would be interesting to see if evidence from eye-tracking corroborates earlier observations on SI with EEG and PET, which reported more activation in the left temporal lobe as well as Broca’s area during L1-to-L2 SI, an indication of higher cognitive load (Kurz, 1995; Tommola et al., 2000).

Researchers also relied on eye-tracking to examine the impact on cognitive load from a variety of problem triggers. For instance, Ma et al.’s (2021) study showed that global cognitive load seemed to be significantly affected by syntactic asymmetry between English and Chinese, as much longer dwell times and more fixations were observed in STR of such structures. In another study, Korpal and Stachowiak-Szymczak (2018) reported that simultaneous interpreting of *numbers* was more cognitively taxing than interpreting of their context, indicated by longer mean fixation duration for numbers. Interpreting strategy is another recurring topic that keeps intriguing researchers. Ma and Li (2021) compared cognitive load associated with two different strategies for English-to-Chinese STR of asymmetric sentences: “reordering” and “chunking.” They identified higher global cognitive load with “reordering,” implied by longer dwell time and more fixation counts, as well as higher local cognitive load with this strategy, signaled by higher rereading rate.

##### Cognitive Processing

Eye-tracking has been applied to capture the often-elusive online processing of interpreting as well, including that of specific problem triggers, in distinct phases, of different groups, and with multi-modal input. In their seminal work, McDonald and Carpenter (1981) examined the processing of ambiguous idiomatic phrases in STR. They observed different fixation patterns between literal and idiomatic translation of these expressions, and argued that STR may be based on normal reading processes, including parsing and error detection. Zheng and Zhou (2018) took a temporal perspective to investigate the processing of metaphorical expressions (ME) in STR with eye-tracking data. Results revealed that the pause prior to targeted ME was not exclusively invested in their processing, and the planning process may have begun before the pause. Some studies have divided interpreting into disparate phases and explored their processing individually. For instance, Chen et al. (2021) innovatively performed semantic gaze mapping and AOI drawing in the note-reading phase of CI, and demonstrated that this process seemed to be a non-linear one. The study also provided support for the usefulness of eye-tracking as a potent visualizing tool for cognitive processing. Studies have also identified processing differences between different groups. Dragsted and Hansen (2009) found that STR processing seemed to be more linear and consecutive for interpreters than translators, who demonstrated more regressions, more fixation counts, and shorter fixation duration. Attempts have also been made to study multi-modal processing in interpreting. Seeber et al. (2020) investigated online processing with both aural and visual information input. By recording eye movements and temporal data, they discovered that in simultaneous interpreting with text, unlike in reading while listening, participants did not look ahead, but instead, showed a visual lag behind aural stimuli. Therefore, they concluded that contrary to previous assumptions, visual input was not utilized to support speech comprehension, but production of output, as fixations on preceding sentences may work to alleviate pressure on short-term memory.

#### Oculomotor Behavior

Another superordinate theme of much investigation is on the physical and external aspect of interpreting: oculomotor behavior *per se*. In our review, 50% of the papers (13) were found to discuss oculomotor behavior with eye-tracking, which allows for granular and real-time investigation of this theme. We extracted 3 sub-themes from them, as can be seen in Figure 5: (1) utilization of visual input (OB-UV); (2) viewing patterns (OB-VP); and (3) the role of gaze (OB-RL).

##### Utilization of Visual Input

Eye-tracking provides valuable information, such as heatmap, fixation duration, and counts for researchers to explore how participants make use of visual input. Wehrmeyer (2014) compared visual attention of hearing and deaf viewers to the screen of sign language interpreted news broadcasts. Heatmaps and fixation analysis showed that hearing viewers’ visual attention was mainly on picture material, and sometimes on subtitles, lip-reading, and the interpreter. For deaf viewers, however, the visual focus was primarily on the interpreter, then on pictorial material, and surprisingly, seldom on subtitles or lip-reading. Thus, this finding called into question the effectiveness of using subtitles as a substitute information source for deaf viewers. In a similar vein, Bosch-Baliarda et al.’s (2020) study sought to ascertain the optimal SLI split screen configuration for deaf viewers to make the most of visual information. It was found that SLI split screen setup did have an impact on information accessibility, and one with the size of 1/4 of the TV screen and in the left position is more favorable to content comprehension.

##### Viewing Patterns

Several attempts have been made to study how viewing patterns are affected by factors such as group difference, directionality, syntactic difficulty, and speech rate. Tiselius and Sneed (2020) compared gaze patterns between experienced and inexperienced dialog interpreters, and noted that the experienced group averted gaze more, especially during interpreting, although the differences were not significant. Directionality also seemed to be in play for gaze patterns, with more gaze aversion in interpreting into the allophone language (mostly L2 in this study) than into L1. This may be due to increased cognitive effort involved in L1-to-L2 interpreting or to the need to keep the turn of the interaction. In another study, a classic professional-trainee comparison was made by Chmiel and Lijewska (2019), who found that trainees viewed source text (ST) less in STR than professionals. This could be because trainee interpreters intentionally looked away to actively avoid interference from source text. In addition, more difficult objective-relative structures lead to less viewing than the easier subjective-relative ones, and this tendency was more prominent for trainees. Korpal and Stachowiak-Szymczak (2020) probed into the effect of speech rate on viewing patterns in SI with slides, and reported more fixation counts per minute at faster rate, indicating scanning instead of careful reading behavior on slides.

##### The Role of Gaze

Several studies have documented the role of gaze from the perspective of pragmatics, and all of them focus on DI. They examined how, in interpreter-mediated triadic interaction, gaze affects backchannel responses, serves as tokens of affiliation similar to head nods, and functions as non-verbal deixis in a similar manner to gestures and head movements (Vranjes et al., 2018, 2019; Vranjes and Brône, 2021).

### Measures

**Figure 6:**
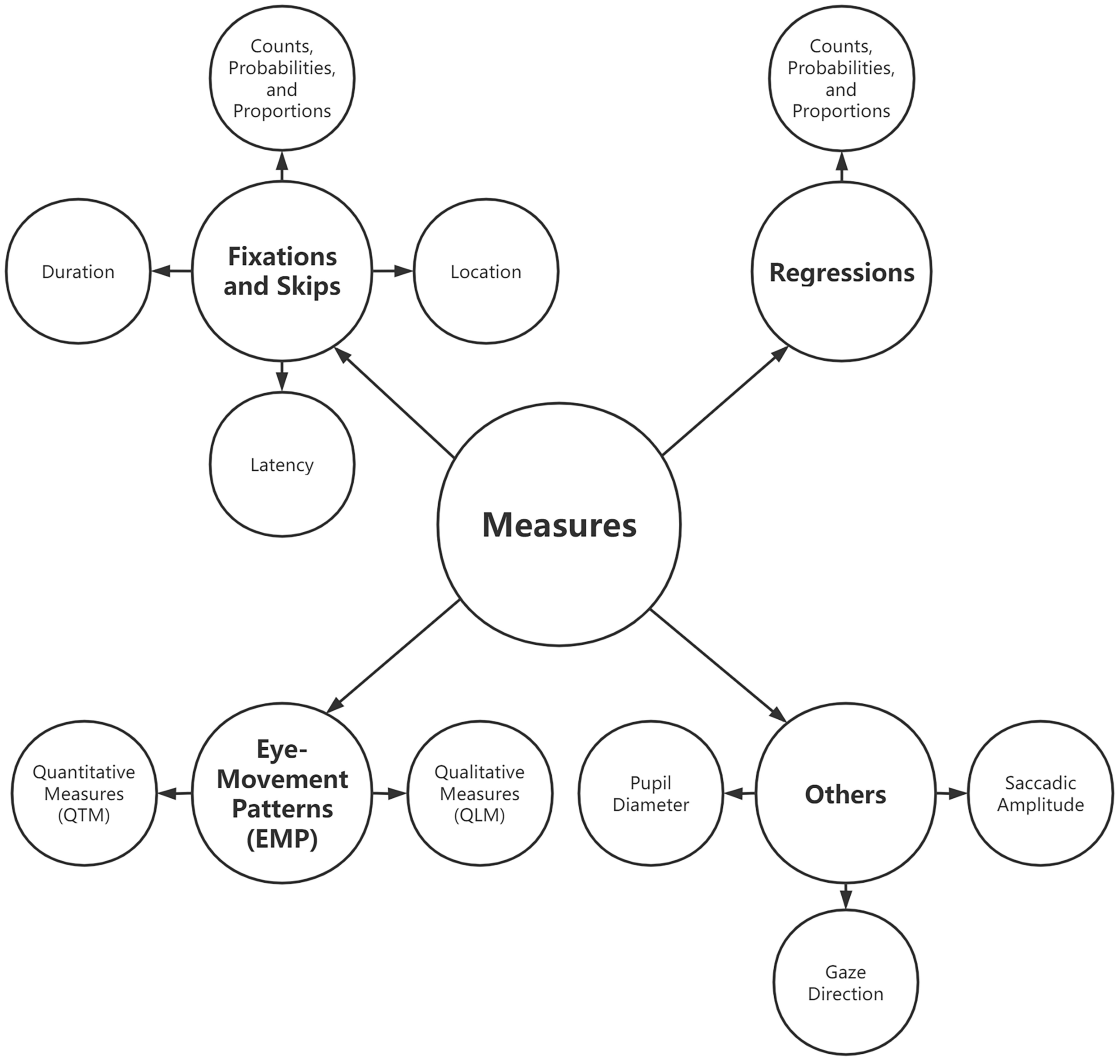
Taxonomy of measures.

**Figure 7:**
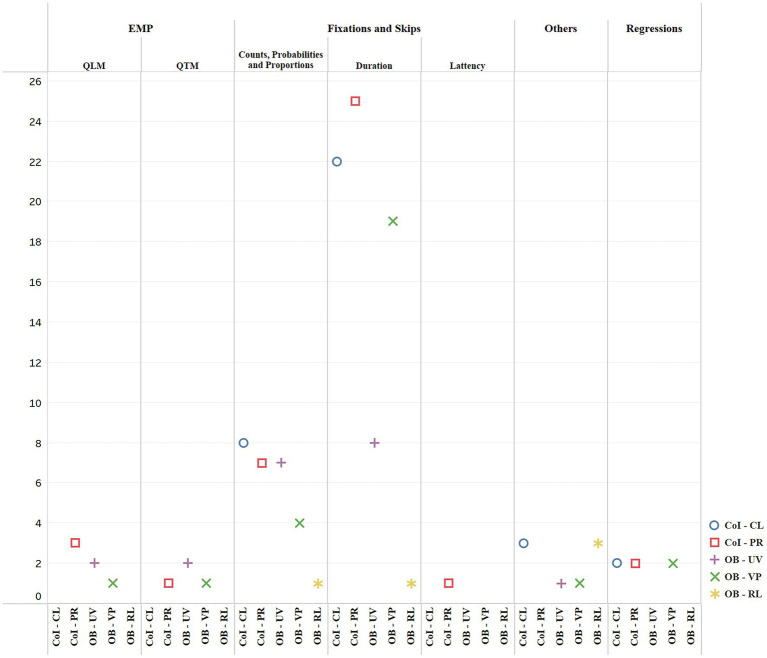
Themes and choice of measures and their frequencies.

One of the strengths of eye-tracking is that it yields a large set of measures (Godfroid and Hui, 2020), and choosing the best-fit measures for analysis is essential to sound eye-tracking studies (Yan et al., 2013). A review of measures employed in the extant literature provides interested researchers with a better sense of common and less common measures in the field. Eye-tracking measures can be classified as early measures, intermediate measures, and late measures according to processing stages (Siyanova-Chanturia, 2013; Conklin et al., 2018). In another widely quoted taxonomy, Radach and Kennedy (2004) categorized them into temporal and spatial measures. This review adopted a taxonomy based on Godfroid (2019) (Figure 6), which is more comprehensive to cover most of the diverse measures used in interpreting studies, including integrated ones such as heatmap and scanpaths. Godfroid (2019) classified measures into three categories: fixation and skips, regressions, and eye movement patterns (EMP). To make it more inclusive for interpreting studies, an extra category of others was added for this review to encompass measures that could not be subsumed to the foregoing three categories, such as pupil diameter and gaze direction. In order to explore how these measures were applied in IS, we then aligned measures with themes, and the results are illustrated in Figure 7.

In general, an average of 4.7 eye-tracking measures were used in IS. As Figure 7 shows, fixations and skips were the most frequently used metrics for both of the two superordinate themes: cognitive investigation and oculomotor behaviors, and they are also most widely used in all of the sub-themes except for exploration into the role of gaze. A closer look revealed that in interpreting studies, this category mostly includes measure of fixations—skips were used only once, therefore, we will limit our discussion to fixations. As one of the two fundamental eye movements, fixation refers to a relatively static state of eyes and usually lasts for 200–300 ms (Rayner, 2009). Its popularity is due to what Just and Carpenter (1980, p. 331) postulate in their immediacy theory: “there is no appreciable lag between what is being fixated and what is being processed.” Within fixations, fixation counts, probabilities and proportions, and duration were the most favored metrics, and they were mainly adopted to examine cognitive processing and cognitive load. This corroborates previous findings, that fixation counts and duration were predominant measures in psycholinguistics and translation studies to indicate cognitive effort (Hvelplund, 2014; Korpal, 2015).

Regressions were often used in the sub-themes of processing and viewing patterns. They are eye movements in the reverse direction of normal reading, such as right-to-left reading in English (Booth and Weger, 2013). Since regressions reflect additional processing of a certain area after the eyes have left it, they are usually indicators of reanalysis (Godfroid, 2019). In the studies reviewed, Ma and Li (2021) found few long-distance regressions when interpreters employed the strategy of “chunking” for syntactic asymmetry in English-to-Chinese STR. They posited that the processing was at a more local level and reading during “chunking” was more continuous than with the strategy of “reordering.” Another example was Chen et al.’s (2021) study, which calculated the regression rate of note-reading in CI. Results showed a rate of 23%, in between that found in reading for comprehension (10–15%) and reading in STR (30–15%) (Rayner, 1998; Shreve et al., 2010). It was thus suggested that note-reading in CI was more linear than reading in STR, but less linear than reading for comprehension.

Eye movement pattern was used to investigate both cognitive processing and oculomotor behavior. It is a type of measure that integrates multiple oculomotor events, and visualizes them into qualitative or quantitative representations. Therefore, they include qualitative measures, such as heatmap and gaze plot, as well as quantitative ones, such as scanpath (Godfroid, 2019). For instance, Su and Li (2020) visualized the preparatory reading phase of STR with scanpath, and found that during this phase of L2-to-L1 STR, interpreters were more of linear readers, while in that of L1-to-L2 STR, more like local readers.

Apart from the above three types, several other measures were employed. For example, pupillometry was put to use to index cognitive load. Surprisingly, however, only one study adopted this measure (Su and Li, 2019) during the past two decades since Hyönä et al. (1995) first demonstrated pupil dilation as a reliable metric for processing effort in IS. Pupil dilation has been used for the investigation of cognitive processes in diverse fields for over a century (Löwenstein, 1920; Kahneman and Beatty, 1966). In addition, a wealth of evidence has shown that more difficult tasks caused larger pupillary response, and demonstrated the reliability of task-evoked pupillary response as a metric for processing load and arousal within tasks, across tasks, and possibly across individuals (Kahneman and Beatty, 1966; Beatty, 1982; Tommola and Niemi, 1986; Siegle et al., 2003; Klingner et al., 2011; Toivo and Scheepers, 2019). Another advantage of pupillometry for indexing cognitive load, as previously mentioned, is that it is more accurate than subjective measures and performance measures, and less intrusive than other psycho-physiological methods (Seeber, 2013). Despite all these advantages, studies in this review tended to prefer fixations over pupil diameter to indicate cognitive load in interpreting. This may be due to various confounding factors for moment-to-moment fluctuations in pupil sizes, including testing-related ones, such as illumination, and participant-related ones, such as fatigue, age, medication, and anxiety level (Eckstein et al., 2017). Therefore, pupil diameter is a more demanding measure to use as researchers have to control for other possible variables. Still, it has great potential in IS, for instance, as a measure to triangulate data from fixations. Novel measures also emerged in the studies reviewed, such as gaze direction and saccadic amplitude. Gaze direction was used to explore the role of gaze, for example, in producing backchannels in DI, such as laughter, head nods, and head shakes (Vranjes et al., 2018). Saccadic amplitude was employed alongside fixations and pupil sizes to indicate cognitive effort (Su and Li, 2019).

One discovery worth noting is that some researchers complemented the foregoing measures with their own tailored measures. For instance, Zheng and Zhou (2018) used mean fixation duration/metaphor word count and total fixation duration/total word count to compare cognitive effort in sight translation of metaphorical expressions and literal expressions, and the study found the former is more cognitively taxing than the latter. Such derived measures are recommended, as this standardization filters out possible confounding variables (Conklin et al., 2018).

Yet in terms of the number of measures, 4 studies used only one measure. For higher confidence in the findings, triangulation, that is, the use of two or more measures or approaches is needed (Heale and Forbes, 2013). Another limitation is that in the data processing of eye-tracking measures, some important information is found missing in the studies reviewed. For instance, 6 of them (23%) did not report data screening criteria, which may impair research replication and thus progress in the field.

## Discussion

Despite their contribution to IS studies, two limitations identified in some studies in this review deserve our attention.

The first one concerns the incomplete reporting of key information about data collection and processing, which undermines data quality and research replicability. For instance, some studies (Dragsted and Hansen, 2009) neglected essential information about eye trackers’ specifications, including sampling rate, precision, accuracy, and latency (Conklin et al., 2018). These four fundamental attributes determine eye-tracking quality, and the right choices concerning them need to be made for the right research. In reading research, for example, eye-tracking systems with a sampling rate of 500 Hz or 1,000 Hz are preferred, and if a low sampling rate (the criteria is under 250 Hz) is adopted for studies with small AOIs, a multitude of data are required to compensate for inaccuracies arising from it (Holmqvist et al., 2011; Conklin et al., 2018). Hence, a detailed account of information about data collection constitutes a basis on which to judge data quality and robustness of the research. Another rationale for increasing transparency in reporting eye trackers’ specifications is that differences in them are found to yield different results. For instance, Leube et al. (2017) found that sampling rate affected saccade detection. More specifically, in a reading task, the 120 Hz mobile eye tracker boasted a significantly higher saccades detection rate and a more accurate estimation of the mean saccade duration than the 60 Hz one. Therefore, reporting of information concerning data collection, including but not limited to eye trackers’ specifications can contribute to research comparability and replicability. In addition, important information on eye-tracking data processing, such as data filter settings, is found missing in some studies (Bosch-Baliarda et al., 2020), and these settings have a direct impact on results. For example, it has been found that effects were significant when inclusion criterion for fixations is set at longer than 140 ms, but insignificant at 50 ms (Yan et al., 2013). Most eye-tracking software allows researchers to decide filter settings, primarily, fixation duration and maximum distance between 2 gaze samples. In order to improve research replicability, information on them needs to be reported, as the ability to replicate findings is crucial to scientific advancement (Open Science Collaboration, 2015).

For these reasons, it is recommended by Godfroid and Hui (2020) that eye-tracking studies should include information on items such as eye trackers (e.g., sampling rate, precision), experiment set-up (e.g., screen size), design (e.g., font size and spacing of the stimuli), data analysis protocol (e.g., data preprocessing), and data quality.

The second limitation is insufficient triangulation, which impairs research validity. Every measure or method has inherent biases, and the adoption of a single approach or measure may lead to premature and erroneous conclusions. Triangulation, a process where researchers seek convergence with multiple data sources and endeavors to offset biases, enhances research validity (Greene et al., 1989; Han, 2018). In the case of eye tracking interpreting research reviewed here, some studies were found to draw a conclusion with a single method of eye-tracking, or a single measure from it. For instance, Korpal and Stachowiak-Szymczak (2018) used mean fixation duration as the sole indicator of cognitive effort. Fixation duration is indeed seen as indicative of cognitive effort (Rayner, 1998; Holmqvist et al., 2011), the use of a single measure, however, is likely to raise doubt on the research results, especially when eye-tracking can yield rich data from a wide array of measures, some of which are also indices of cognitive load, such as fixation count and pupil size. In addition, Korpal and Stachowiak-Szymczak (2018) used the single method of eye-tracking to extrapolate cognitive load on participants. However, this method has its share of weaknesses despite various merits. Eye movements are not the impeccable representation of cognitive mechanisms, because oftentimes cognitive shifts can happen independent of eye movements, as is illustrated by the phenomenon of wandering or drifting (Shepherd et al., 1986; Smallwood and Schooler, 2006). Therefore, eye-tracking is believed to reflect a “proximation of the relationship between visual focus and cognitive focus” (Hvelplund, 2014, p. 209). In light of this situation, it will be most potent when data obtained is cross-checked and supplemented by those yielded from other methods. Therefore, Korpal and Stachowiak-Szymczak’s (2018) study would have been more convincing if results from eye-tracking on cognitive load were confirmed by other methods, such as stimulated recall (Seeber, 2013). In summary, in order to strengthen validity, multiple eye-tracking measures need to be triangulated with other online measures such as ERP, or off-line measures, such as questionnaires, interpreting product analysis, and stimulated recall.

Based on our analysis, in future research, eye-tracking should generate increased scholarly attention on the investigation of multimodality in interpreting, especially for interpreting modes whose multimodal feature has traditionally been neglected. The necessity to process multimodal information is seen as one of the defining characteristics of interpreting (Seeber, 2017), but eye-tracking has primarily been applied to examine text-reading in sight translation, and future research will see more study into multimodal processing in DI, SI, SLI, and CI for the following reasons. Firstly, with technological development, more affordable, flexible and mobile eye trackers with higher precision would come along (Cognolato et al., 2018). This makes it easier to probe, for example, how interpreters deal with both nonverbal messages and speech of the speakers in DI, and how interpreters handle both text and speech in SI with text. Moreover, with the popularity of automatic speech recognition and of distance interpreting in the wake of COVID-19, interpreters are more likely to be faced with multimodal input than before. It is not uncommon that service providers transfer speech to text and project them on screen real time for simultaneous interpreters and audience (Li, 2020), thereby offering them both aural and visual information. In light of these new trends, Pöchhacker (2019) identified intermodality as one of the five novel features of interpreting. International Association of Conference Interpreters (2019) also issued guidelines on distance interpreting in 2019, where they recommended large and clear enough screens for interpreters to see texts, images, speakers, participants, and the conference room clearly. Against this backdrop, growing popularity of applying eye-tracking into the study of multimodal interpreting processing is likely.

In addition, it would be interesting to investigate with eye-tracking how interpreting processes differ between different language pairs and between long and short segments. Studies can also be conducted to examine the processing of interpreting abbreviations, enumerations, idiomatic expression, and accented and technical speech. Furthermore, eye-tracking has great potential for interpreting testing. Recent years has seen an increasing number of studies that used eye-tracking to evaluate L2 reading, listening, and speaking assessment, and they mainly focused on test validity (Conklin and Pellicer-Sánchez, 2016; Godfroid and Hui, 2020). Similarly, eye-tracking can be a powerful tool for interpreting assessment. For example, researchers can compare online eye movements of disparate groups, such as professional interpreters and interpreting trainees, for the same test. The results provide insight into the discriminating power of this test, in other words, test validity. Lastly, in future studies, saliency approach could be adopted in IS with eye-tracking and contribute to the cross-fertilization of both disciplines of IS and computer science. As a data analysis method that can be used to predict eye movement patterns like spatial location of individual fixations and their sequential order (Foulsham and Underwood, 2008), it has a wide variety of models that are extensively applied (Borji et al., 2013), but it is rarely adopted in the studies we review. Saliency models goes beyond simply describing eye movements by predicting where interpreters might look, thus deepening our understanding of interpreting. Additionally, since interpreting is a complex activity (Hale, 2010; Stachowiak-Szymczak, 2020), results from IS with eye tracking constitute valuable information for comparison between and evaluation of the performance of different saliency models (He et al., 2019).

## Conclusion

The purpose of the current review was to synthesize papers on IS with eye-tracking so as to depict a comprehensive and systematic picture of their contributions, limitations and implications, as well as future directions. We have found that in general, comparison of different participant groups, in particular, expert-novice comparison, instead of focusing on a single group is the most popular design. We have argued for the recruitment of extra participants, given the recurring data loss in eye-tracking, and cautioned against overgeneralizing findings to interpreters on the whole, when only trainees are enrolled for investigation. In addition, STR is the most popular mode for examination. When it comes to the choice of eye trackers, remote ones are the most preferred type, and it is necessary to adopt the right eye tracker in alignment with research questions, so as to achieve a balance between ecological, external validity, and reliability. This review has also shown that eye-tracking is used for two strands of research: cognitive investigation and observation of oculomotor behaviors. The former theme includes evaluation of cognitive load and exploration of cognitive processing, and the latter covers topics of utilization of visual input, viewing patterns, and the role of gaze. As far as eye-tracking measures are concerned, we have noted that fixations were the most favored metrics for all of the foregoing themes apart from the study into the role of gaze. Researchers are also found to adopt novel measures, such as saccadic amplitude to index cognitive effort, as well as tailored measures for standardization. We have argued that in order for researchers to build on each other’s work and for scientific progress, detailed reporting of key information on data collection and processing, such as sampling rate of eye trackers, set-up information and data filtering criteria are necessary for future studies. Furthermore, the results would be more convincing with more triangulation of multiple measures and methods. In terms of future work, it would be interesting to see how more advanced eye-tracking systems can offer new insight into the multimodal processing of previously underexplored interpreting modes, such as SI, CI, DI, and SLI. Further research could also be conducted to determine the interpreting processing of different language pairs, speech of different lengths, abbreviations, enumerations, idiomatic expression, and accented and technical speech. Another interesting research agenda would be to investigate how eye-tracking can be applied in interpreting testing, especially in the examination of test validity. It is also recommended that saliency approach be applied in future studies. One major limitation of this study needs to be acknowledged. We included only peer-reviewed empirical papers and English publications for the review. Notwithstanding this limitation, the present study, as the first attempt at a comprehensive review of IS with eye-tracking, should prove valuable for practitioners, educators, and researchers alike. Findings reported here will offer insight into the nature of interpreting, have pedagogical implications for interpreting educators, and inform researchers’ efforts in using eye-tracking in conducting interpreting research.

## Author Contributions

TH is responsible for the conceptualization, design, data collection and analysis, and writing of the manuscript. XW is responsible for the conceptualization, data collection and analysis, visualization, and writing of the manuscript. HX is responsible for the conceptualization, design, and suggestions of revisions. All authors contributed to the article and approved the submitted version.

## Funding

This study was funded by Zhejiang Conservatory of Music (G001A3032033).

## Conflict of Interest

The authors declare that the research was conducted in the absence of any commercial or financial relationships that could be construed as a potential conflict of interest.

## Publisher’s Note

All claims expressed in this article are solely those of the authors and do not necessarily represent those of their affiliated organizations, or those of the publisher, the editors and the reviewers. Any product that may be evaluated in this article, or claim that may be made by its manufacturer, is not guaranteed or endorsed by the publisher.
