# Supplementary material for: Eye-Tracking in Interpreting Studies: A Review of Four Decades of Empirical Studies
Source: Front Psychol. 2022 Jun 27;13:872247. doi: 10.3389/fpsyg.2022.872247 (PMC9272778; doi:10.3389/fpsyg.2022.872247)
Supplement: Supplementary file 2 [file Table_2.docx]

**Appendix. Summary of included empirical articles**

| **Author(s)(Year)** | **Findings and contribution** | **Mode** | **Participants** | **Eye tracker and mode** | **Theme(s)** | **Measure(s)** |
| --- | --- | --- | --- | --- | --- | --- |
| Bosch-Baliarda et al. (2020) | A screen configuration with a SL interpreter screen of medium size (1/4 of the TV screen width) and on the left improved content access for deaf sign language users. | SLI | Deaf viewers(n=32) | Tobii T60, remote | UV/VP | Fixation count, mean fixation duration, visit count, mean visit duration, heat map |
| Chen (2020) | L1-to-L2(Chinese-English) CI induced lower cognitive load in the comprehension phase and more cognitive effort in the production phase. | CI | Professional interpreters(n=26) | SMI ETG, head-mounted | CL | Average fixation duration |
| Chen et al. (2021) | 1.Note-reading in CI seemed to be a non-linear process; 2. Note-taking choices of Phase I (comprehension and note-taking) of CI affect the cognitive processing in note-reading during Phase II (note-reading and production) | CI | Professional interpreters(n=26) | SMI ETG, head-mounted | VP/CL | Number of fixations, average fixation duration, gaze mapping, first fixation duration first-pass dwell time second-pass dwell time total dwell time, number of revisits skip rate |
| Chmiel and Lijewska (2019) | 1.Professionals were more efficient than trainees; 2. Trainees viewed the source text less than professionals, possibly to avoid source text interference, especially when processing more difficult syntax;3. More difficult object-relative sentences took longer to translate, but triggered less viewing than the easier subject-relative sentences. | STR | Professional interpreters(n=24) vs. Interpreting trainees(n=15) | EyeLink 1000+, remote | CP/VP | Gaze duration, regression path duration, total duration, percentage of dwell time |
| **Author(s)(Year)** | **Findings and contribution** | **Mode** | **Participants** | **Eye tracker and mode** | **Theme(s)** | **Measure(s)** |
| Dragsted and Hansen (2009) | Interpreters' processing during STR seemed to be more manner than that of translators, and fundamental behavioral differences were found between translators and interpreters in written and sight translations. | STR | Professional interpreters(n=4) vs. Professional translators(n=4) | Tobii | CP/VP | Hotspot analyses, (total) fixation count, average fixation duration |
| Ho (2021) | 1. Compared with trainees, professionals showed better language flexibility and their interpreting was characterized by higher accuracy, better steadiness, a faster speed, less disruption by pauses;2. Professionals adopted more diverse approaches at the beginning of STR. | STR | Professional interpreters(n=18) vs. Interpreting trainees(n=18) | EyeLink 1000 | CP/VP | Number of fixations, mean fixation duration, first fixation duration, gaze duration, go-past time, re-reading time, total viewing time |
| Ho et al. (2020) | 1.Counterintuitively, interpreting trainees demonstrated higher efficiency than bilinguals in STR, not because read faster, but because they were more “economical” in reading; 2. Trainees’ superior performance could be attributed to chunking strategy. | STR | Interpreting trainees(n=18) vs. Bilinguals(n=18) | EyeLink 1000 | CP/VP | Number of fixations, mean fixation duration, first fixation duration, gaze duration, go-past time, re-reading time, total viewing time |
| Hyönä et al.(1995) | This methodological study lends support to the use of pupillary response as an effective indicator of processing load during simultaneous interpretation and other language tasks. | SI | Interpreting trainees(n=27) | Applied Science Laboratories eye-tracker Model 1994, unspecified | CL | Pupil size |
| **Author(s)(Year)** | **Findings and contribution** | **Mode** | **Participants** | **Eye tracker and mode** | **Theme(s)** | **Measure(s)** |
| Korpal and Stachowiak-Szymczak (2018) | 1. In SI of numerical and its context, professionals achieved higher accuracy than trainees; 2. Numerical and its context SI accuracy was positively correlated; 3. SI of numerical was more cognitively demanding than interpreting their context. | SI | Professional interpreters(n=30) vs. Interpreting trainees(n=25) | EyeLink 1000+, remote | CL | Mean fixation duration |
| Korpal and Stachowiak-Szymczak (2020) | 1. The rate of source speech had an impact on accuracy of number interpreting on both professionals and trainees; 2. When interpreting speech with a fast pace, more scanning, instead of careful reading of the slides presented was observed in participants; 3. Professionals achieved higher accuracy in interpreting of numbers than trainees, regardless of source speech rate. | SI | Professional interpreters(n=30) vs. Interpreting trainees(n=24) | EyeLink 1000+, remote | UV | Fixation count per minute (on slides), fixation count per minute (on numbers), percentage of gaze time to numbers |
| Lozano-Argüelles et al. (2020) | Apart from in the two L2 groups (English learners of Spanish) for CV paroxytone words, all of the three participants groups predicted word suffixes with suprasegmental and segmental information on lexical stress and syllable structure. Furthermore, both L2 groups predicted later than monolingual L1(Spanish) users, but interpreters achieved higher predictive efficiency compared with non-interpreters in almost all conditions, and with monolinguals in CV oxytones and CVC paroxytones. | Not specified | Professional interpreters(n=22) vs.  L2 learners (n=25) vs. Monolingual L1 users(n=25) | EyeLink 1000+, head-stabilized | CP | Rate and proportion of target fixations |
| **Author(s)(Year)** | **Findings and contribution** | **Mode** | **Participants** | **Eye tracker and mode** | **Theme(s)** | **Measure(s)** |
| Ma and Li (2021) | To deal with word-order asymmetry, 1) the strategy of *chunking* was used far more frequently than *reordering* regardless of the presence of contextual information, 2) when provided with contexts, higher cognitive load was observed, 3) reordering triggered more cognitive load, both globally and locally. | STR | Interpreting trainees(n=23) | EyeLink 1000+, head-stabilized | CP/CL | Dwell time, fixation count, rereading rate, gaze plots |
| Ma et al. (2021) | 1. Word order asymmetry exerted a significant effect on global cognitive load; 2. The existence of context mitigated impact from the asymmetry, but only to a limited extent; 3. Most participants preferred reordering to segmentation for sight translating such asymmetrical structure, despite a higher load on working memory induced by the former strategy. | STR | Interpreting trainees(n=25) | EyeLink 1000+, head-stabilized | CL | Dwell time and fixation count, first fixation duration, regression path duration |
| Marschark et al. (2005) | Counterintuitively, the amount that participants learned from interpreted(signed) lecture, in other words, their comprehension of sign language, was not affected by if there were three-dimensional visuospatial cues or student-interpreter feedback. This may suggest that in college classroom, video-based interpreting can be as effective as real-life interpreting. | SLI | Students (ss) with no knowledge of sign language (n=10) vs. Deaf ss who were skilled singers (n=11) vs. Deaf ss who were new signers (n=11) | Custom-built, head-mounted | UV | Gaze direction, total gaze duration, mean gaze duration |
| **Author(s)(Year)** | **Findings and contribution** | **Mode** | **Participants** | **Eye tracker and mode** | **Theme(s)** | **Measure(s)** |
| McDonald and Carpenter (1981) | This paper investigated the processing of ambiguous idiomatic phrase in STR, and suggested a model for interpretation, parsing and error recovery, which was found to be based on normal reading processes. | STR | Translators(n=2) vs. Bilinguals(n=2) | Applied Science Laboratories eye tracker, unspecified | CP | Initial reading pass, translation pass, error recovery pass, forward fixations, regressions |
| Seeber et al. (2020) | In SI with text, instead of looking ahead, interpreters exhibited a visual lag behind aural stimuli. Visual input mainly served to support production of output, not speech comprehension. | SI | Professional interpreters(n=15) | EyeLink 1000, remote | CP | Proportion of fixations, mean dwell time |
| Stachowiak-Szymczak and Korpal (2019) | 1.Trainees relied on visual aids-slides, more than professionals in the interpreting of numbers, as they exhibited longer fixations on slides; 2. Numbers seemed to induce higher cognitive effort in trainees than professionals; 3. Trainees’ SI of numbers was less accurate than professionals; 4. Access to slides improves the accuracy of number renditions. | SI | Professional interpreters(n=26) vs. Interpreting trainees(n=22) | EyeLink 1000+, remote | CL/UV | Fixation count, mean fixation duration, total gaze time |
| Su and Li (2019) | The study found that STR directionality exerted an effect on cognitive load and problem triggers. More specifically, L1-to-L2(Chinese-English) STR was more cognitively demanding than the other direction. In this direction, punctuation mark and the head-final noun phrase constituted major problem triggers, in contrast with those in the opposite direction, such as less frequent words and head-initial noun phrase. | STR | Interpreting trainees(n=9) | Tobii TX300, remote | CP/CL | Fixation duration, total number of fixations, pupil dilation, saccadic amplitude, heat map, scanpath, visit duration, first fixation |
| **Author(s)(Year)** | **Findings and contribution** | **Mode** | **Participants** | **Eye tracker and mode** | **Theme(s)** | **Measure(s)** |
| Su and Li (2020) | In rehearsed STR, 1) participants invested considerably more time, but less cognitive effort in preparatory reading than in actual sight interpreting, 2) directionality affected the processing of both these two stages, for instance, in preparatory reading, participants were more of linear readers in L2-to-L1 (English-Chinese) STR, but more of local readers in the opposite direction. | STR | Interpreting trainees(n=14) | Tobii TX300, remote | CP/CL | Average fixation duration, reinspective fixation duration, scanpath |
| Su and Li (2021) | Training contributes to better processing efficiency and better output quality in rendition of both the whole text and such problem trigger as low frequency words in STR, but such advantage was not found in the STR of complex noun phrases. Moreover, no greater training advantage was found in L2-to-L1(English-Chinese) direction than the more difficult opposite direction, potentially due to ceiling effect. | STR | Beginner interpreting trainees(n=10) vs. Advanced interpreting trainees(n=8) | Tobii TX300, remote | CL | Mean fixation duration, total fixation duration |
| Tiselius and Sneed (2020) | During L1-to-L2 DI, interpreters gazed at no face more often, in other words, averted gaze more, potentially indicative of higher cognitive load in this direction. Moreover, there were not significant differences in gaze patterns between experienced and inexperienced interpreters. | DI | Experienced interpreters(n=7) vs. Inexperienced interpreters(n=10) | SMI Glasses 2.0, head-mounted | VP/CL | Mean number of fixations |
| **Author(s)(Year)** | **Findings and contribution** | **Mode** | **Participants** | **Eye tracker and mode** | **Theme(s)** | **Measure(s)** |
| Vranjes and Brône (2021) | This study examined how interpreters used gaze, along with other semiotic resources such as gesture and head movements, to differentiate their own voice and the voice of the speaker, whose speech they were rendering. Two kinds of strategies were employed by interpreters: 1) multimodal layering-pointing or gaze shift was accompanied by verbal quotative, and 2) nonverbal layering-these embodied gestures were used without verbal quotative. | DI | Professional interpreters(n=6) & Speakers(n=12) | ArringtonGig-E60 and Tobii, head-mounted | RL | Gaze direction |
| Vranjes et al. (2018) | This study delved into the role of gaze in producing backchanneling in interpreter-mediated dialogue, such as continuers, acknowledgements, and newsmarkers. It found that interpreters were crucial in sustaining a triadic participation framework. | DI | Professional interpreters(n=1) & Speakers(n=2) | Pupil Pro Binocular Glasses and Arrington Gig-E60, head-mounted | RL | Gaze direction, number of gaze fixations |
| Vranjes et al. (2019) | The study found that in interpreter-mediated therapeutic talk, differences in the interpreter’s and therapist’s affiliative listener responses were potentially reflective of their different roles and involvement. | DI | Professional interpreters(n=1)& Speakers(n=2) | Pupil Pro Binocular Glasses and Arrington Gig-E60, head-mounted | RL | Gaze duration, gaze direction |
| **Author(s)(Year)** | **Findings and contribution** | **Mode** | **Participants** | **Eye tracker and mode** | **Theme(s)** | **Measure(s)** |
| Wehrmeyer (2014) | The study found that deaf viewers and hearing viewers utilized visual input from sign language interpreted news programs differently. Specifically, hearing viewers’ visual attention was mainly on picture material, and sometimes on subtitles, lip-reading and the interpreter. Deaf viewers, however, focus primarily on the interpreter, and seldom on subtitles or lip-reading. | SLI | Deaf viewers(n=13) vs. Hearing viewers(n=20) | Tobii T60, remote | UV/VP | Mean fixation duration, total visit duration for each AOI, total fixation durations as a percentage of video time, Heat map |
| Zheng and Zhou (2018) | 1.For most participants, the time of the preceding pause was not invested exclusively in reading and processing the targeted metaphorical expressions (ME) alone;2. When sight translating an ME, the planning step seemed to have happened prior to the pause before the targeted ME; 3.STR of MEs was more cognitively taxing than that of literal expressions. | STR | Translation trainees(n=24) | Tobii TX300, remote | CP/CL | Fixation duration distribution, (standardized) fixation duration |
